# Structural insight into conformational change in prion protein by breakage of electrostatic network around H187 due to its protonation

**DOI:** 10.1038/s41598-019-55808-1

**Published:** 2019-12-17

**Authors:** Juhwan Lee, Iksoo Chang

**Affiliations:** 1 Center for Proteome Biophysics, DGIST, Daegu, 42988 Korea; 2 Department of Emerging Material Sciences, DGIST, Daegu, 42988 Korea; 3 Core Protein Resources Center, DGIST, Daegu, 42988 Korea; 4 Supercomputing Bigdata Center, DGIST, Daegu, 42988 Korea; 5 Department of Brain and Cognitive Sciences, DGIST, Daegu, 42988 Korea

**Keywords:** Biophysics, Computational biophysics, Protein analysis

## Abstract

A conformational change from normal prion protein(PrP^C^) to abnormal prion protein(PrP^SC^) induces fatal neurodegenerative diseases. Acidic pH is well-known factors involved in the conformational change. Because the protonation of H187 is strongly linked to the change in PrP stability, we examined the charged residues R156, E196, and D202 around H187. Interestingly, there have been reports on pathological mutants, such as H187R, E196A, and D202N. In this study, we focused on how an acidic pH and pathological mutants disrupt this electrostatic network and how this broken network destabilizes PrP structure. To do so, we performed a temperature-based replica-exchange molecular dynamics (T-REMD) simulation using a cumulative 252 μs simulation time. We measured the distance between amino acids comprising four salt bridges (R156–E196/D202 and H187–E196/D202). Our results showed that the spatial configuration of the electrostatic network was significantly altered by an acidic pH and mutations. The structural alteration in the electrostatic network increased the RMSF value around the first helix (H1). Thus, the structural stability of H1, which is anchored to the H2–H3 bundle, was decreased. It induces separation of R156 from the electrostatic network. Analysis of the anchoring energy also shows that two salt-bridges (R156-E196/D202) are critical for PrP stability.

## Introduction

Prion diseases are fatal neurodegenerative diseases caused by misfolding of prion protein (PrP)^1–3^. The three main causes of prion diseases are infection, genetic mutations, and unknown reasons related to sporadic disorders^4,5^. These three factors lead to PrP destabilization, which induces a conformational change from the normal cellular isoform (PrP^C^) to a misfolded isoform (PrP^SC^). Oligomeric forms of PrP^SC^ have neurotoxic and infective properties^6^. Several fatal neurodegenerative diseases, such as Creutzfeldt-Jakob disease, and Gerstmann-Sträussler-Scheinker disease, are linked to oligomeric forms of PrP^SC^.

*In vivo*, the conformational change from PrP^C^ to PrP^SC^ occurs in the endocytic pathway, whose internal lysosomes have a low pH^7,8^. Additionally, many previous *in vitro* studies showed that the environmental pH condition is critical to PrP misfolding and aggregation^9^. In particular, the pH-dependent PrP^SC^ oligomer transition occurs at pKa ~4.7^10,11^. This suggests that histidine protonation may play a key role in PrP stability. Titration experiments and proton-exchange rates were used to measure the pKa of H187, which is a structurally buried and sequentially highly conserved histidine. The pKa value is dramatically downshifted to ~5 in H187^12^. In contrast, the other histidine residues have pKa values between 6.5 and 7. Additionally, *in vivo* experiments involving H187R mutant, which mimics the wild-type PrP with a protonated H187, also revealed increased misfolding and oligomerization^13,14^. These studies showed that the protonation of H187 is a critical factor in the dramatic destabilizing of the PrP structure, which promotes misfolding and oligomerization.

**Figure 1:**
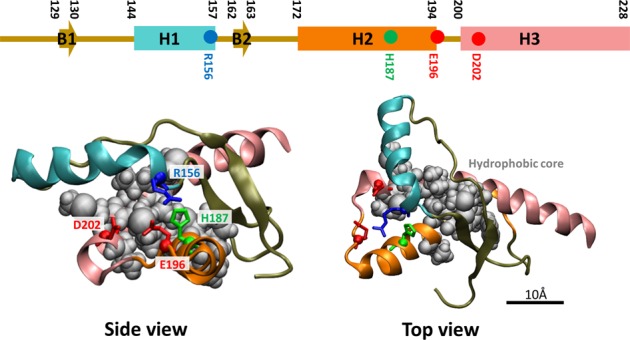
Normal PrP structure. The C-terminal globular domain of PrP contains three α-helices H1 (cyan), H2 (orange), and H3 (pink) and two β-sheets B1 and B2. Side and top views of the normal PrP structure. Charged residues in the electrostatic network (R156, H187, E196, and D202) are represented by the licorice and C_α_ balls, and the hydrophobic core in the H2–H3 bundle is shown with gray balls.

The conformational change in PrP is a physicochemical process at the molecular level. Folding and misfolding states of PrP are determined by the free energy difference and the height of the energy barrier between the folding and misfolding states. In many protein systems, hydrogen bonds and strong salt bridges enforce specific spatial configurations of charged residues^15,16^. The electrostatic interaction is one of the most critical contributors to the free energy value and height of the energy barrier. Under acidic pH conditions, a change in the protonation state of H187 could disrupt the electrostatic balance of the surrounding area. Thus, in the present study, we focused on the charged residues R156, E196, and D202, which are located within 7 Å of H187 (Fig. 1). The NMR structure of human PrP shows that the protonation of H187 can destabilize the R156–E196 salt bridge and that D202 is close enough to interact with R156 and H187^3^. Interestingly, pathological mutants of H187, E196, and D202 (but not R156) have also been reported. The H187R mutant was reported in the classical form of Gerstmann-Sträussler-Scheinker in an American family^17,18^, the E196A mutant has been associated with Creutzfeldt-Jakob disease^19^, and D202N was linked to atypical Gerstmann-Sträussler-Scheinker with Alzheimer’s disease-like phenotypes^20^. Thus, these pathological mutants affect the electrostatic balance, which is critical for the structural stability of PrP.

In terms of the electrostatic network on the PrP structure, PrP consists of an unstructured flexible N-terminal tail and C-terminal globular domain, which has three conserved α-helices (H1, H2, and H3) and two β-sheets (B1 and B2). Positively charged R156 (located in H1) strongly interacts with negatively charged E196 and D202 (located in H2 and H3). These interactions prevent dissociation of H1 from the H2–H3 bundle. However, protonated H187 is subject to a repulsive force from R156 that interrupts the R156–E196/D202 salt bridge. As a result, H1 can be separated from the H2–H3 bundle, allowing exposure of the hydrophobic core between H2 and H3 to water^21,22^. This exposed hydrophobic core critically decreases the structural stability of PrP.

To calculate the stability of PrP, we used temperature-based replica-exchange molecular dynamics (T-REMD), which is the most popular computational method to calculate thermodynamic and kinetic properties. It is useful for enhancing ensemble sampling across high-energy barriers and mapping free energy. Previous T-REMD studies of PrP were run under highly unstable conditions, involving 9 protonated residues and a high temperature (temperature range, 300 to 500 K) to observe the β-rich conformation of PrP^SC^^23,24^. The work successfully obtained the β-rich conformation but missed atomic details in the electrostatic network around H187. In this study, we characterized the electrostatic network of wild-type and mutant PrP (R156A, H187R, E196A, and D202N) with a structured C-terminal domain according to the protonation state of H187 and temperature (temperature range, 300.00 to 360.81 K).

Through our T-REMD simulations, we greatly increased the conformational ensemble sampling. We measured the atomic distance between charged residues to determine the spatial configuration of the electrostatic network. Additionally, we calculated the root mean square fluctuation (RMSF) and anchoring strength of R156 to compare the structural stabilities of PrP isoforms. We found that the electrostatic network is disrupted by pathological mutants and that these pathological mutants are critically affected by acidic conditions.

## Results

### The spatial configuration of wild-type PrP under neutral pH conditions

**Figure 2:**
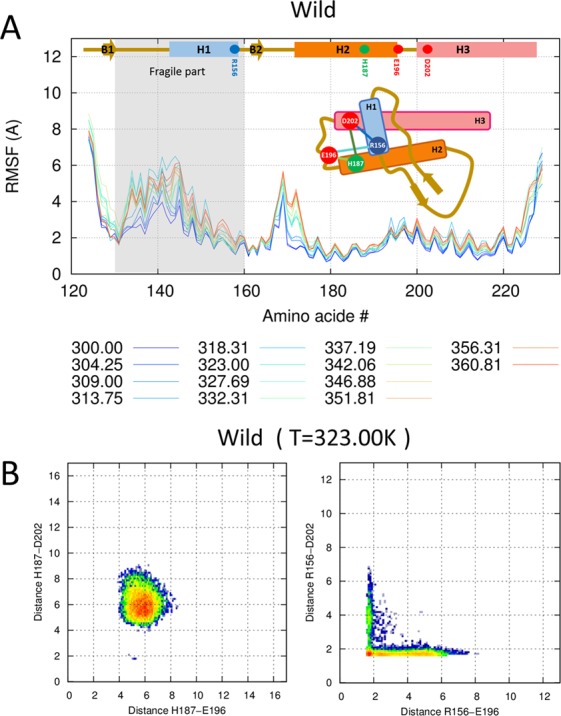
Wild-type PrP stability at neutral pH. (**A**) RMSF values are shown for all temperatures with the color gradient ranging from blue (300.00 K) to red (360.81 K). (**B**) Conformational free energy heat maps (in arbitrary units) calculated from the H187–E196/D202 distance population (left panel) and the R156–E196/D202 distance population (right panel) at 323.00 K.

To determine the structural stability of PrP, we assessed the atomic fluctuations according to temperature. The fluctuation change showed that the most thermodynamically fragile part is residues 130–160, which contain H1 and the surrounding loop part (Fig. 2A). This result is in accordance with that of a previous NMR relaxation measurement study, which found that H2 and H3 form a relatively rigid core and that H1 is more flexible than the other two helixes^25^. However, H1 tightly holds the H2–H3 bundle through charge interaction. We calculated the conformational free energy from the ensemble population. The free energy heat map for the minimum distances of H187–E196/D202 at 323.00 K revealed that H187 keeps the stable distance with E196/D202 (left panel of Fig. 2B and SFig. 1A). In the case of the R156–E196/D202 minimum distance, the heat map has an L-shape with a red dot on the edge (right panel of Fig. 2B and SFig. 1B). The L-shape means that at least one salt bridge between R156 and E196/D202 is formed in all configurations, and the red dot represents the two strong salt bridge configurations between R156 and E196/D202. This charge interaction network maintains the thermodynamic stability of H1, H2, and H3. Thus, the hydrophobic core in H2 and H3 is maintained by wrapped H1.

### Environmental pH conditions affect the electrostatic network

**Figure 3:**
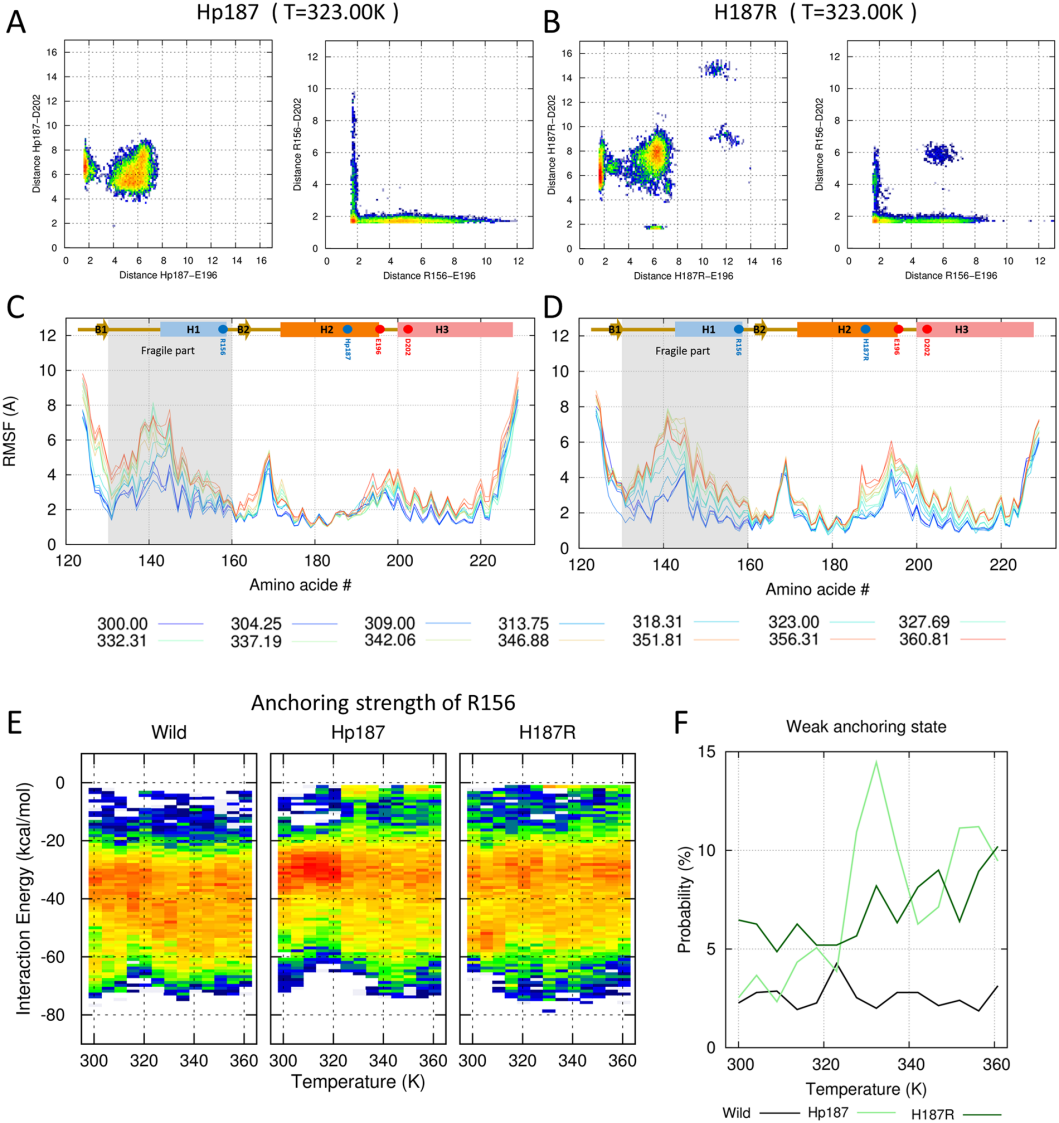
Effects of pH on PrP. (**A**,**B**) Conformational free energy heat maps (in arbitrary units) calculated from the populations of the Hp187(H187R)–E196/D202 distance (left panels of A and B) and the R156–E196/D202 distance (right panels of A and B). (**C**,**D**) RMSF values are shown for all temperatures with the color gradient ranging from blue (300.00 K) to red (360.81 K) at 323.00 K. (**A**,**C**) The effect of the acidic environment is mimicked by Hp187. (**B**,**D**) The effect of the protonated histidine is mimicked by arginine mutation. (**E**) Distributions of anchoring strengths for wild-type and Hp187/H187R are calculated over the temperature range. (**F**) The probabilities of a weak anchoring state for wild-type and Hp187/H187R are shown with the black and light-green/dark-green lines.

The effect of an acidic environment was mimicked using protonated H187 (named Hp187) and H187R mutant. The minimum distance between Hp187(H187R) and E196 was decreased in acidic environment simulations, forming a strong left-side basin in the free energy heat map (left panels of Fig. 3A,B and SFigs. 2A and 3A). Two positively charged residues, R156 and Hp187(H187R), compete for one negatively charged residue E196. As a result, the R156–E196 distance is increased, with a longer horizontal line in the right panels of Fig. 3A,B (SFigs. 2B and 3B). Using the long side chain of arginine, H187R occasionally makes a salt bridge with D202, forming the bottom side basin in the left panel of Fig. 3B. Because H187R and R156 are also competing for D202, the charge interactions of R156–E196 and R156–D202 simultaneously become weak (blue island in the right panel of Fig. 3B).

To compare the anchoring strength of R156, we calculated the van der Waals, electrostatic, and polar solvation energies between R156 and the H2–H3 bundle (residues 171–228), which are major contributors to the free energy barrier. The anchoring energy ranges from about –20 to –60 kcal/mol (left panel of Fig. 3E), and the most probable anchoring energy is –32.6 kcal/mol. This strong electrostatic interaction creates a high free energy barrier between the folded and misfolded conformations. Under acidic conditions, local basins appear in the 0 to –15 kcal/mol region (middle and right panels of Fig. 3E). Thus, we defined the weak anchoring state using a –15-kcal/mol cutoff. Figure 3F shows that the weak anchoring probability starts to increase from 320 K. This result contrasts with wild-type PrP at a neutral pH, which has a stable anchoring structure at all simulation temperatures. Under neutral pH conditions, the weak anchoring probability is ~3% (maximum, 4.3%) at all temperatures, but the probability increases up to 14.5% in Hp187 (10.2% in H187R, Fig. 3F). The PrP structure has a 17.6 kcal/mol energy gap between the cutoff for the weak anchoring state (–15 kcal/mol) and anchoring energy minima (–32.6 kcal/mol). This energy gap will act as an energy barrier to the conformational change. To overcome this energy gap, the fragile part could be fully extended. The extension length of PrP is ~100 Å in the model structure (SFig. 10). One previous *in vitro* study of the energy barrier used force spectroscopy measurements (FSMs) and determined an energy barrier of 15.2 ± 1.4 kcal/mol and an extension length of 90 ± 10 Å^26^.

Additionally, the maximum fluctuation of the fragile part is increased from 6.5 Å in wild-type PrP to 8.1 Å in Hp187 (7.9 Å in H187R) and the C terminus of H2 and the loop between H1 and H2 are destabilized in Fig. 3C,D^27^. These results successfully explain the *in vitro* results obtained using hydrogen-deuterium exchange coupled with mass spectrometry (HDX-MS)^28,29^. Their results showed that H1, the loop between H1 and B2, and the loop between H2 and H3 are destabilized under acidic pH conditions.

### The protonated histidine (Hp187) critically affects the thermostability of the E196A mutant

**Figure 4:**
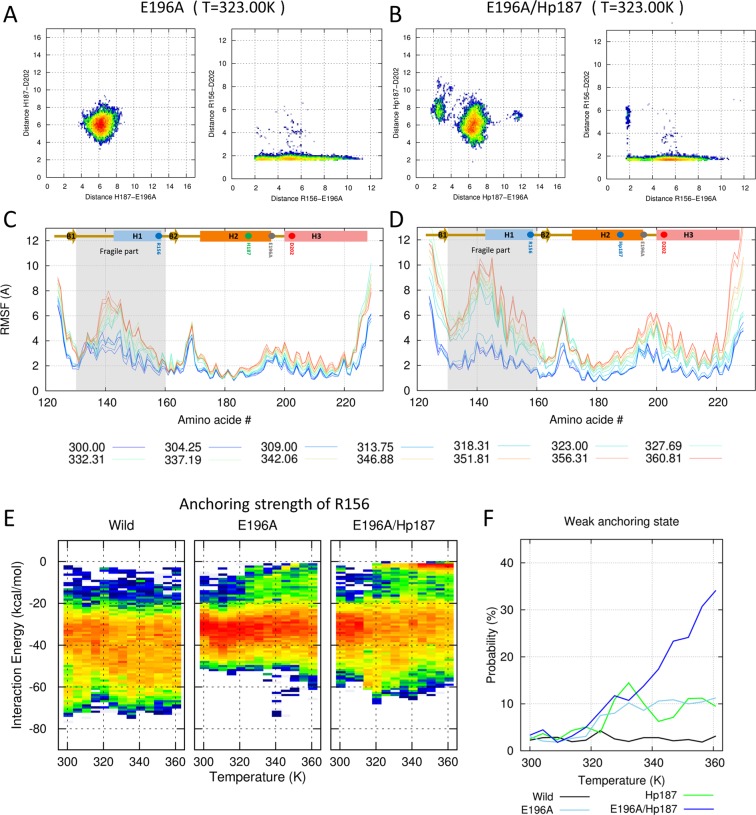
Effects of the E196A mutant. (**A**,**B**) Conformational free energy heat maps (in arbitrary units) calculated from the populations of the H187(Hp187)–E196A/D202 distance (left panels of A and B) and the R156–E196A/D202 distance (right panels of A and B) at 323.00 K. (**C**,**D**) RMSF values are shown for all temperatures with the color gradient ranging from blue (300.00 K) to red (360.81 K). (**A**,**C**) Effects of the E196 mutant under neutral pH conditions. (**B**,**D**) Effects of the E196 mutant under acidic pH conditions. (**E**) Distributions of anchoring strengths for wild-type, E196, and E196A/Hp187 are calculated over the temperature range. (**F**) The probabilities of weak anchoring states for wild-type, E196, and E196A/Hp187 are shown with black and light-blue/dark-blue lines.

We broke the R156–E196 salt bridge using the E196A mutant to determine the role of this salt bridge. The free energy heat map showed that the distribution of the H187–E196A/D202 minimum distance was similar to that of wild-type PrP (left panel of Fig. 4A and SFig. 4A). However, the interaction between R156 and E196A became weak. Nonetheless, the R156–D202 salt bridge was itself more stabilized to compensate for the R156–E196 interaction and maintain the anchored conformation of R156. Thus, the red dot on the edge split into two small basins and the vertical line disappeared in the R156–E196/D202 heat map (right panel of Fig. 4A and SFig. 4B). The effect on the fragile part looked similar to the Hp187 condition (Fig. 4C).

The E196A/Hp187 mutant mimics the E196A mutant under acidic conditions. The minimum distance between Hp187 and E196A is decreased and a new left-side basin is formed in the left panel of Fig. 4B (SFig. 5A). In contrast, the R156–E196A distance increases. The two small basins in the right panel of Fig. 4A (SFig. 4B) merged to form a large block in the right panel of Fig. 4B (SFig. 5B). In addition, a change in the electrostatic network decreased the structural stability of PrP. The RMSF value is increased in the whole region, and the effect on the fragile part is particularly critical (Fig. 4D). Dramatic changes were also evident in anchoring energy. The range of anchoring energy shrinks to –20 to –45 kcal/mol in both E196A and E196A/Hp187 (Fig. 4E), and the probability of weak anchoring states is increased as temperature increases. Interestingly, the E196A/Hp187 mutant gets an additional red basin in the weak anchoring region (right panel of Fig. 4E). The weak anchoring probability also increases by 34% in the E196A/Hp187 mutant (Fig. 4F).

In wild-type PrP, the average pair-wise interaction energies of R156–E196 and R156–D202 were –9.9 kcal/mol and –22.8 kcal/mol, respectively. These findings differ from the previous energy calculation^30^. In the previous work, the R156–E196 interaction was stronger than the R156–D202 interaction. However, the previous work only used NMR structures to the energy calculation, which only had a salt bridge in R156–E196. Additionally, the results are insufficient to explain other *in vitro* findings. Mutation of D202 has a more dramatic effect than that of E196^31^. We overcame the sampling problem using our prolonged T-REMD simulation and managed to get fully equilibrated configurations that have two stable salt bridges in R156–E196 and R156–D202.

The two strong interactions of R156–E196 (–9.9 kcal/mol) and R156–D202 (–22.8 kcal/mol) are important for the structural stability of PrP, but the main interaction for the anchored conformation is R156–D202. The role of R156–E196 is to safeguard and support the anchoring conformation. Without this safeguard, R156 maintains the anchoring conformation in favorable environments, such as at a low temperature and neutral pH. However, the PrP structure becomes easily unstable under high temperature and acidic conditions when the safeguard is removed, as in the E196A mutant^31,32^.

### D202N mutant induces a weakly anchored conformation

**Figure 5:**
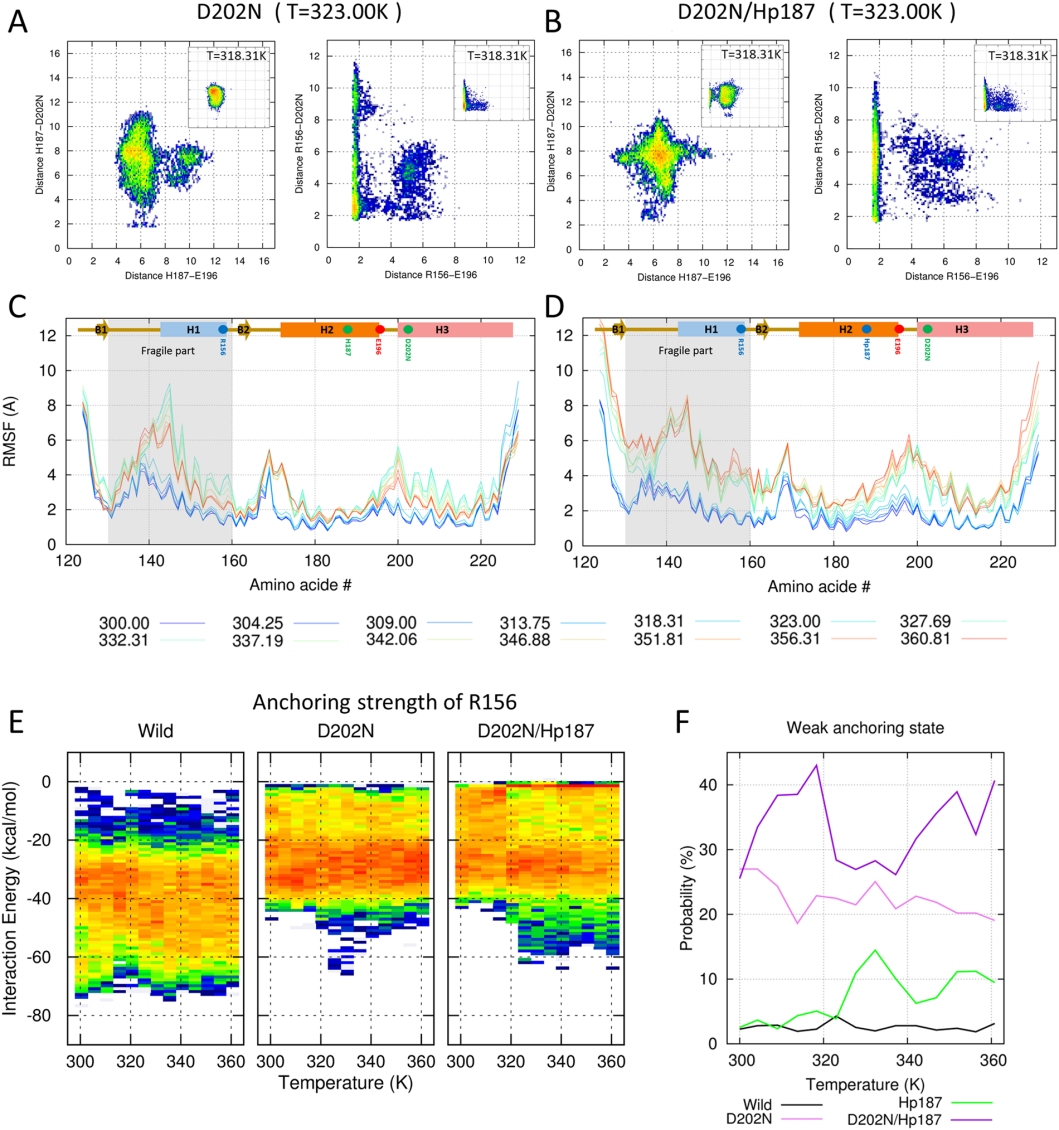
Effects of the D202N mutant. (**A**,**B**) Conformational free energy heat maps (in arbitrary units) calculated from the populations of the H187(Hp187)–E196/D202N distance (left panels of A and B) and the R156–E196/D202N distance (right panels of A and B) at 323.00 K (at 318.31 K for the inset figure). (**C**,**D**) RMSF values are shown for all temperatures with the color gradient ranging from blue (300.00 K) to red (360.81 K). (**A**,**C**) Effects of the D202N mutant under neutral pH conditions. (**B**,**D**) Effects of the D202N mutant under acidic pH conditions. (**E**) Distributions of anchoring strengths for wild-type, D202N, and D202N/Hp187 are calculated over the temperature range. (**F**) The probabilities of weak anchoring states for wild-type, D202N, and D202N/Hp187 are shown with black and pink/purple lines.

The largest contribution to the anchoring energy is the R156–D202 salt bridge. We disrupted this salt bridge using the D202N mutant. Figure 5A,B and the inset figure show the minimum distance heat maps from 323.00 K and 318.31 K. The typical change in the heat maps was seen above 323.00 K. Distance increases are observed in the H187–E196 (left panel of Fig. 5A and SFig. 6A) and R156–D202N pairs (right panel of Fig. 5A and SFig. 6B), and the red dot at the edge becomes weak in the left panel of Fig. 5A (SFig. 6B). The D202N mutant shows a more dispersed shape and does not have a strong basin in 323.00 K compared with E196A. This means that the electrostatic network in the D202N mutant is more thermally unstable than in the E196A mutant. The fluctuation increases not only the fragile part, but also the loop region between H2 and H3 in D202N (Fig. 5C).

The D202N/Hp187 mutant mimics the D202N mutant under acidic conditions. The salt bridge between Hp187 and E196 is present at 318.31 K but is diminished at 323.00 K (left panel of Fig. 5B and SFig. 7A). Additionally, the electrostatic interaction of R156–D202N is more destabilized, with the basin shifted to the large R156–D202N distance value in the right panel of Fig. 5B (SFig. 7B). The RMSF value is increased in the whole region, as in the E196A/Hp187 mutant (Fig. 5D). Significant changes are shown in the anchoring energy. The yellow band is extended to the 0 kcal/mol region in both D202N and D202N/Hp187. The free energy basin, which represents the red line at –0.9 kcal/mol, is shown for D202N/Hp187 (Fig. 5E). The probability of weak anchoring states is greatly increased in all temperature ranges (Fig. 5F).

These dramatic changes in the interaction network reduce the energy barrier between the folding and misfolding states and thus can increase misfolding and oligomerization rates. The misfolding and oligomerization rates of PrP mutants have previously been studied using far-UV circular dichroism (CD) and size-exclusion chromatography (SEC). Our results showed that misfolding and oligomerization rates are more drastically affected by the R156–D202 than the R156–E196 salt bridge. Additionally, thermostability is dramatically decreased in the D202N mutant (T_m,wild-type_ = 63.2 °C, T_m,D202N_ = 54.2 °C at pH 4)^31^.

### The separation of alanine (R156A) from the electrostatic network

**Figure 6:**
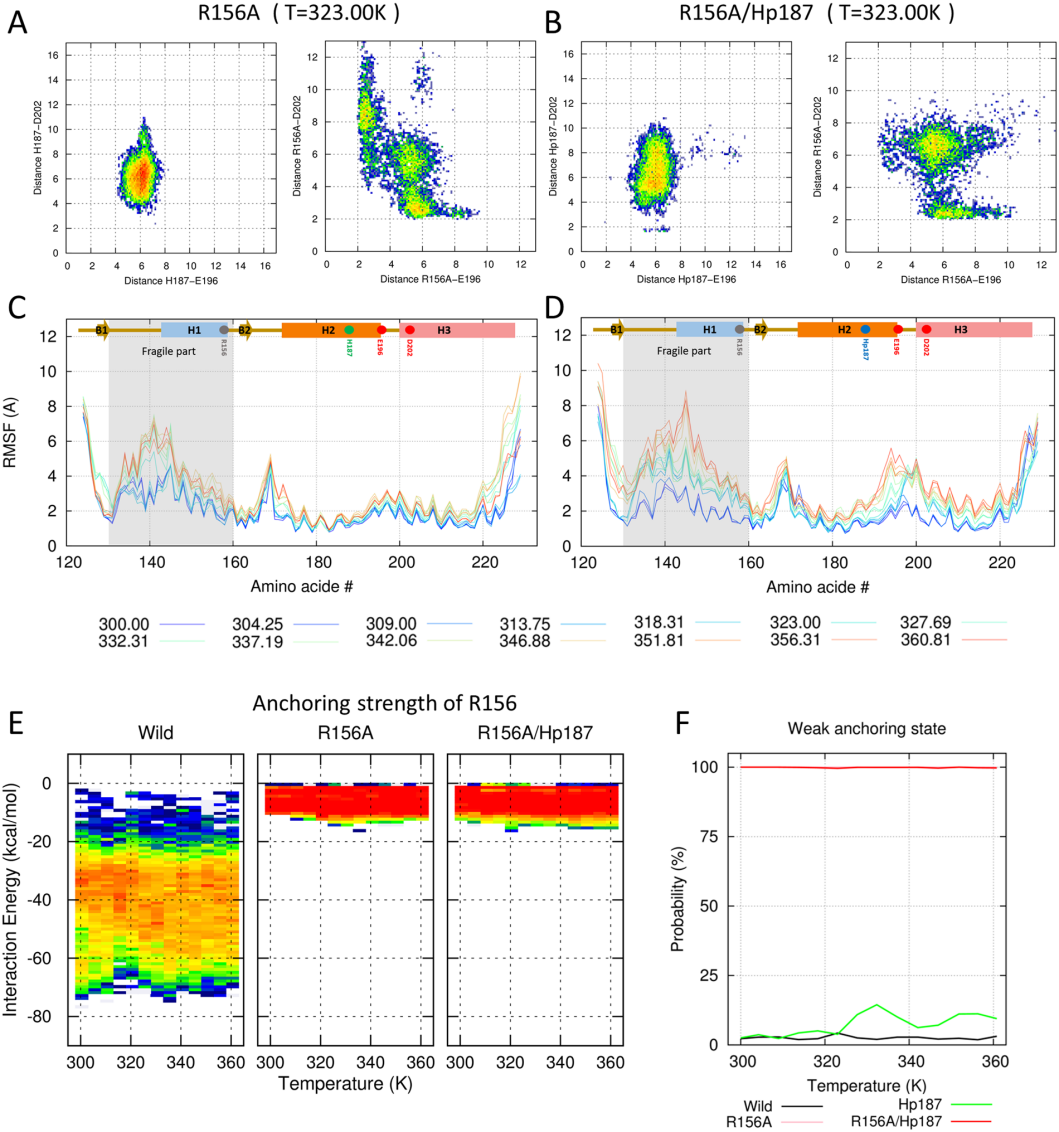
Effects of the R156A mutant. (**A**,**B**) Conformational free energy heat maps (in arbitrary units) calculated from the populations of the H187(Hp187)–E196/D202 distance (left panels of A and B) and the R156A–E196/D202 distance (right panels of A and B) at 323.00 K. (**C**,**D**) RMSF values are shown for all temperatures with the color gradient ranging from blue (300.00 K) to red (360.81 K). (**A**,**C**) Effects of the R156A mutant under neutral pH conditions. (**B**,**D**) Effects of the R156A mutant under acidic pH conditions. (**E**) Distributions of anchoring strengths for wild-type, R156A, and R156A/Hp187 are calculated over the temperature range. (**F**) The probabilities of weak anchoring states for wild-type, R156A, and R156A/Hp187 are shown with black and pink/purple lines.

Using the R156A mutant,we were able to simultaneously break the two salt bridges at R156–E196 and R156–D202. H187 maintained the electrostatic balance with E196/D202 at 323.00 K (left panel of Fig. 6A and SFig. 8A). Because of the short side chain of alanine and the loss of the charge state, R156A never has a hydrogen bond with both E196 and D202 at the same time (right panel of Fig. 6A and SFig. 8B). Thus, the fluctuation is increased in the fragile part (Fig. 6C).

**Figure 7:**
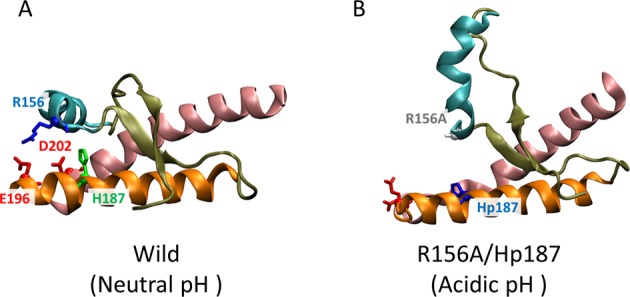
Two snapshots from wild-type and R156A/Hp187. (**A**) R156 is stably anchored to the H2–H3 bundle in wild-type PrP, but (**B**) shows the separation of R156A from the electrostatic network in the R156A/Hp187 mutant.

The R156A/Hp187 mutant mimics the R156A mutant under acidic conditions. The spatial configuration on histidine is no significant difference between R156A and R156A/Hp187 mutants (left panel of Fig. 6B and SFig. 9A). The most notable change is that R156A cannot form a stable hydrogen bond with E196 (right panel of Fig. 6B and SFig. 9B). Figure 7 is a snapshot from wild-type and R156A/Hp187 PrP. It shows the separation of R156A from the electrostatic network. The weak electrostatic network increases the fluctuation in the fragile part and the loop region between H2 and H3 (Fig. 6D). Additionally, heat maps for the anchoring energy markedly shrink, so that almost all configurations show weakly anchored conformations in both R156A and R156A/Hp187 (Fig. 6E,F). These results explain the previous CD and SEC studies^31^. The misfolding and oligomerization rates increase depending on the charge state of R156. Additionally, a HDX-MS study showed that deuterium incorporation rates were increased in H1, the loop between H1 and H2, and the loop between H2 and H3^31^. Those findings are in line with our RMSF results.

## Discussion

The key residue that induces the conformational conversion of PrP under acidic pH conditions is H187. The electrostatic network around H187 stabilizes the PrP structure. R156, H187, E196, and D202 are members of this electrostatic network. Pathological mutant studies of the electrostatic network have been reported^33^. However, to understand the effect of the pathological mutants on the electrostatic network, we used T-REMD, which is a modern method to enhance ensemble sampling. Through a total simulation time of 252 μs, we obtained various structural ensembles.

Here, we measured the thermodynamic properties of wild-type PrP. The RMSF was increased in the fragile part (residues 130–160) and a helical core consisting of H2 and H3 stabilized the structure at all temperature examined (Fig. 2A). A previous NMR relaxation measurement study also found that the rigid core of the H2 and H3 helices, which is stabilized by an inter-helix disulfide bond, and the other parts, including H1, are relatively flexible^25^. These results are consistent with the amyloid fibril-like structure of PrP^SC^. Many electron microscopy and atomic force microscopy studies of amyloid fibrils reported that left-handed β-helices are the key element of amyloid fibrils^34^. Interestingly, a conformational change occurs in PrP sequence residues 89–175 from PrP^C^ to the β-helical conformation of PrP^SC^, and the H2 and H3 structure is maintained, with the inter-helix disulfide bond retained^35^. The increased misfolding and oligomerization rates at low pH also destabilize the fragile part.

We also determined the effect of the pathological mutant on the electrostatic network. The effect of low pH on the electrostatic network is that protonated histidine Hp187 acts as an obstructer at the R156–E196 salt bridge. A mutation at E196 also breaks the R156–E196 salt bridge. Interestingly, the effect of E196A mutant is more dramatic in acidic environments. The thermostability is decreased and the anchoring energy becomes weaker as the temperature increases. As the anchoring energy becomes smaller, H1 more easily separates from the H2–H3 bundle. Previous HDX-MS studies revealed that the B1–H1–B2 domain (residues 132–167) is dissociated from the H2–H3 bundle (residues 174–230) before oligomerization^28^. FSM studies detected three different misfolding pathways during the refolding process, which starts from the extended structure, in single-molecule approaches^36^. Thus, CD and SEC experiment results show that the transition temperature decreases and the misfolded fraction increases according to the charge state of E196^31^.

The interaction energy of R156–D202 was twice as strong as that of the R156–E196 pair. The effect on the electrostatic network was greater in the D202N mutant. Thus, the misfolding and oligomerization rates are more drastically increased and melting temperature decreased by 9.0 °C (T_m,wild-type_ = 63.2 °C, T_m,D202N_ = 54.2 °C at pH 4)^31^. The pKa of D202 is 3.7, calculated from the NMR structure^37^. This means that both H187 and D202 can be protonated at pH 3.7 or less. Under high acidic conditions, Dp202 breaks the electrostatic network and reduces the anchoring energy, as in the D202N mutant. Interestingly, the pH-induced misfolding transition in H187F mutant occurs at pH 3.8^31^. Phenylalanine does not alter the charge state at pH 5. Thus, the H187F mutant can maintain a stable PrP structure until D202 is protonated.

Since we found that two salt bridges were removed in the R156A mutant and severely damages the electrostatic network, it would be interesting to check in the future experimental study whether the R156A mutant is pathogenic or not. This information can be used in drug design^38^. For example, tegobuvir, which is used as a hepatitis C drug, is a recently identified compound with anti-prion activity. Because tegobuvir docks close to the electrostatic network, it strongly interacts with R156, Q160, H187, and K194^39,40^. It thus stabilizes the conformational fluctuation of PrP^C^.

## Methods

Extensive sampling is a basic requirement for the accurate calculation of thermodynamic and kinetic properties. The energy barrier between the local minima is the main hurdle faced by proteins traversing the energy landscape. To overcome energy barriers and enhance ensemble sampling, we used the T-REMD method. This method allows extensive sampling across the local minima based on system temperature exchanges. We used a narrow temperature interval between the replicas (~4.3 K) for a sufficient exchange ratio^41^.

Human PrP (hPrP) consists of the flexible N-terminal tail (residues 23–124) and C-terminal globular domain (residues 125–230). It has been known that NMR structures of C-terminal globular domain of the recombinant hPrP(23–230) and two C-terminal fragments hPrP(90–230), hPrP(121–230) are very similar^3^, although each of which has the different N-terminal length. The secondary structures of C-terminal globular domain of hPrP(23–230), hPrP(90–230), and hPrP(121–230) coincide in three α-helices (residues 144–154, 173–194 and 200–228) and two β-sheet (residues 128–131 and 161–164)^3^. In our simulation, the initial configuration of the three-dimensional structure for residues 125–228 (PDB code: 1QLX) was NMR structure of the recombinant hPrP which is cloned in Escherichia coli (residues 23–230)^3^. All simulations were performed with AMBER18 simulation package with ff14SB force field^42^, and the TIP3 water model^43^ was used. The particle-mesh Ewald (PME) method was used for long-range electrostatic interactions^44^, and a 9 Å distance cutoff was applied for short-range and nonbonded interactions. The bond length of hydrogen atoms was constrained by the SHAKE algorithm^45^. The temperature was controlled by Langevin dynamics with a γ = 2.0 collision frequency^46,47^. Before the production was run, we performed 4,000 steps of energy minimization. During the 100 ps heating process, the system temperature was gradually increased to the target temperature. After the heating step, we applied a 1 ns side chain equilibration time under a 0.5 kcal/mol restraint on the protein backbone. In the NVT ensemble, the T-REMD production run was performed with 14 replicas (300.00, 304.25, 309.00, 313.75, 318.31, 323.00, 327.19, 332.31, 337.19, 342.06, 346.88, 351.81, 356.31, and 360.81)^48^. The cumulative simulation time was 252 μs (2 μs for each replica and 9 types of simulations). The time step was 2 fs, and replica exchanges were attempted every 100 ps. The final 189 μs ensemble (the last 1.5 μs ensemble for each replica) was used for analysis with cpptraj tools, which are provided in the AMBER program package^49^. The structural figure was made with VMD software^50^.

## Supplementary information

Supplementary information
