# Supplementary material for: Structural insight into conformational change in prion protein by breakage of electrostatic network around H187 due to its protonation
Source: Sci Rep. 2019 Dec 17;9:19305. doi: 10.1038/s41598-019-55808-1 (PMC6917724; doi:10.1038/s41598-019-55808-1)
Supplement: Supplementary file 1 — Supplementary information [file 41598_2019_55808_MOESM1_ESM.docx]

**SFigure 1.**


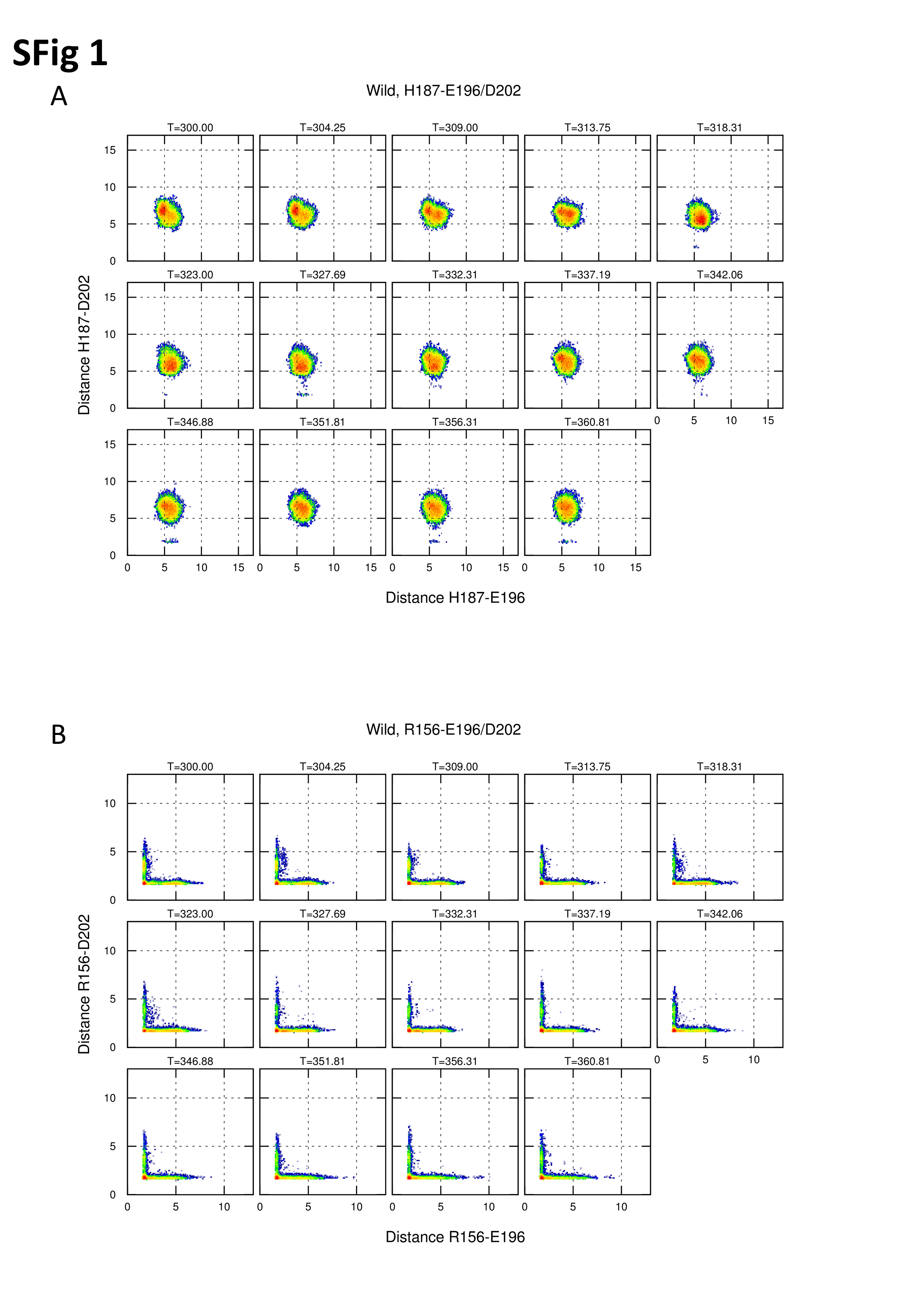


**SFig 1.** Wild-type conformational free energy heat maps (in arbitrary units) calculated from the populations of the H187–E196/D202 distance (A) and R156A–E196/D202 distance (B) at all temperatures.

**SFigure 2.**


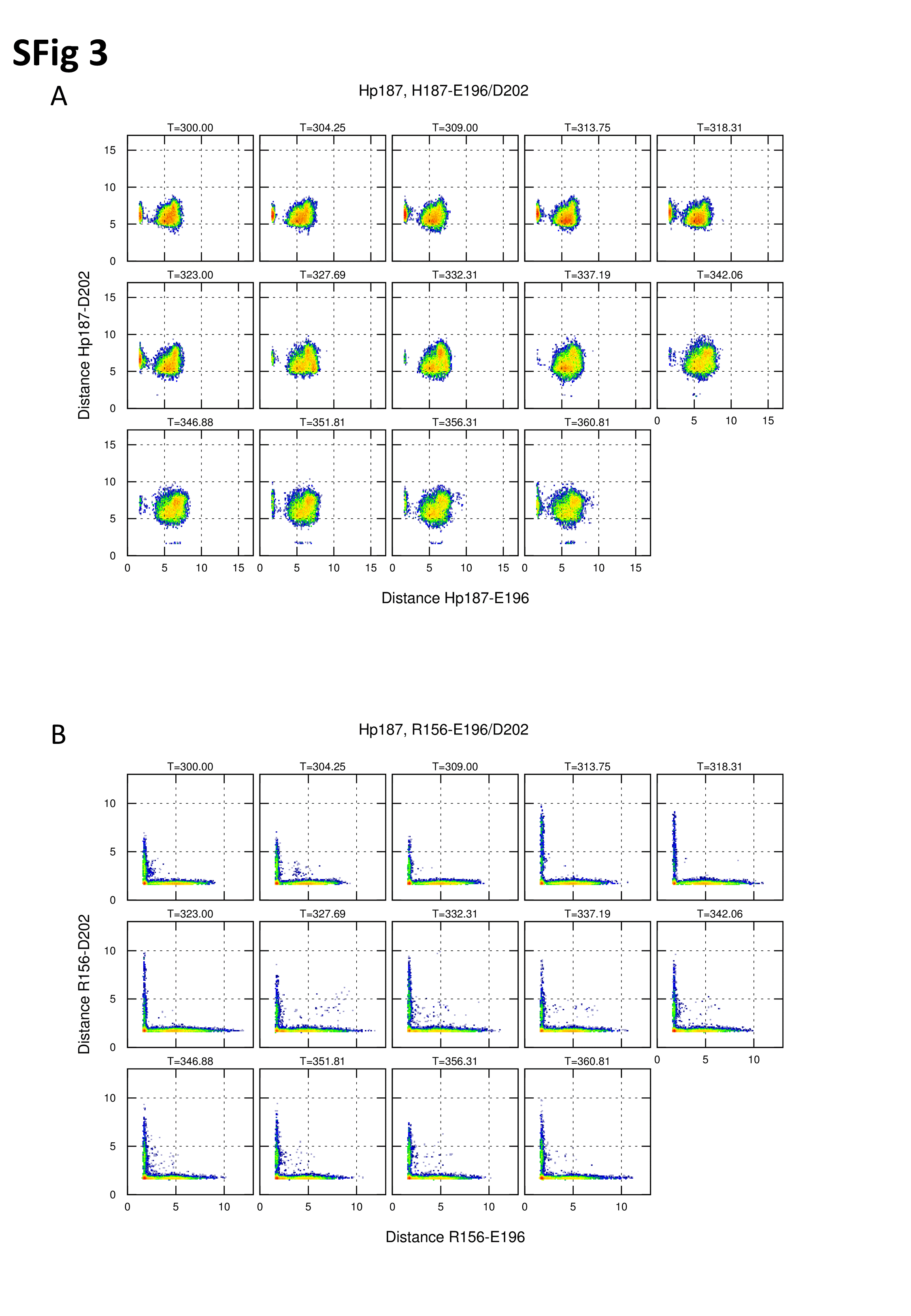


**SFig 2.** Hp187 conformational free energy heat maps (in arbitrary units) calculated from the populations of the H187–E196/D202 distance (A) and R156A–E196/D202 distance (B) at all temperatures.

**SFigure 3.**


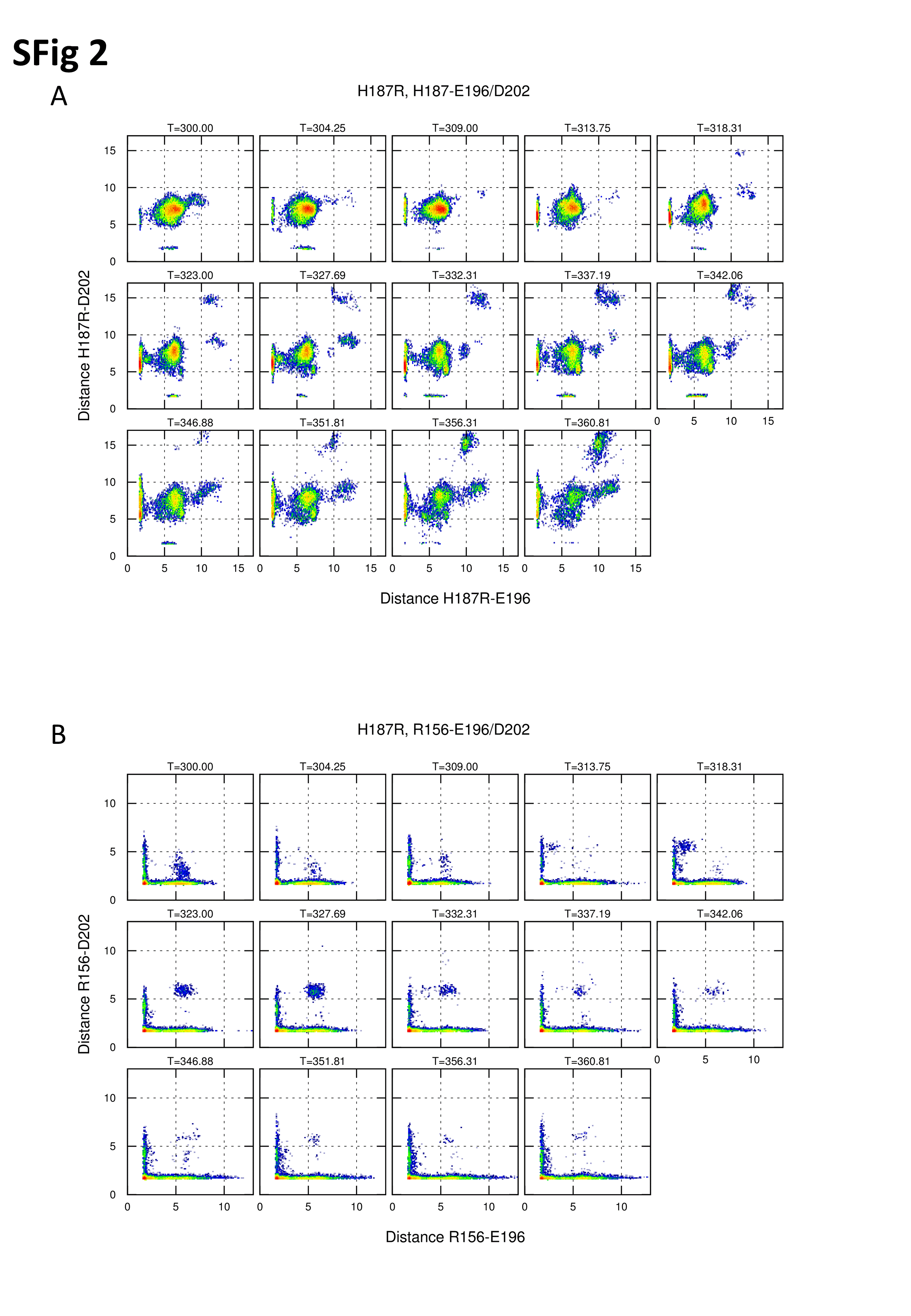


**SFig 3.** H187R mutant conformational free energy heat maps (in arbitrary units) calculated from the populations of the H187–E196/D202 distance (A) and R156A–E196/D202 distance (B) at all temperatures.

**SFigure 4.**


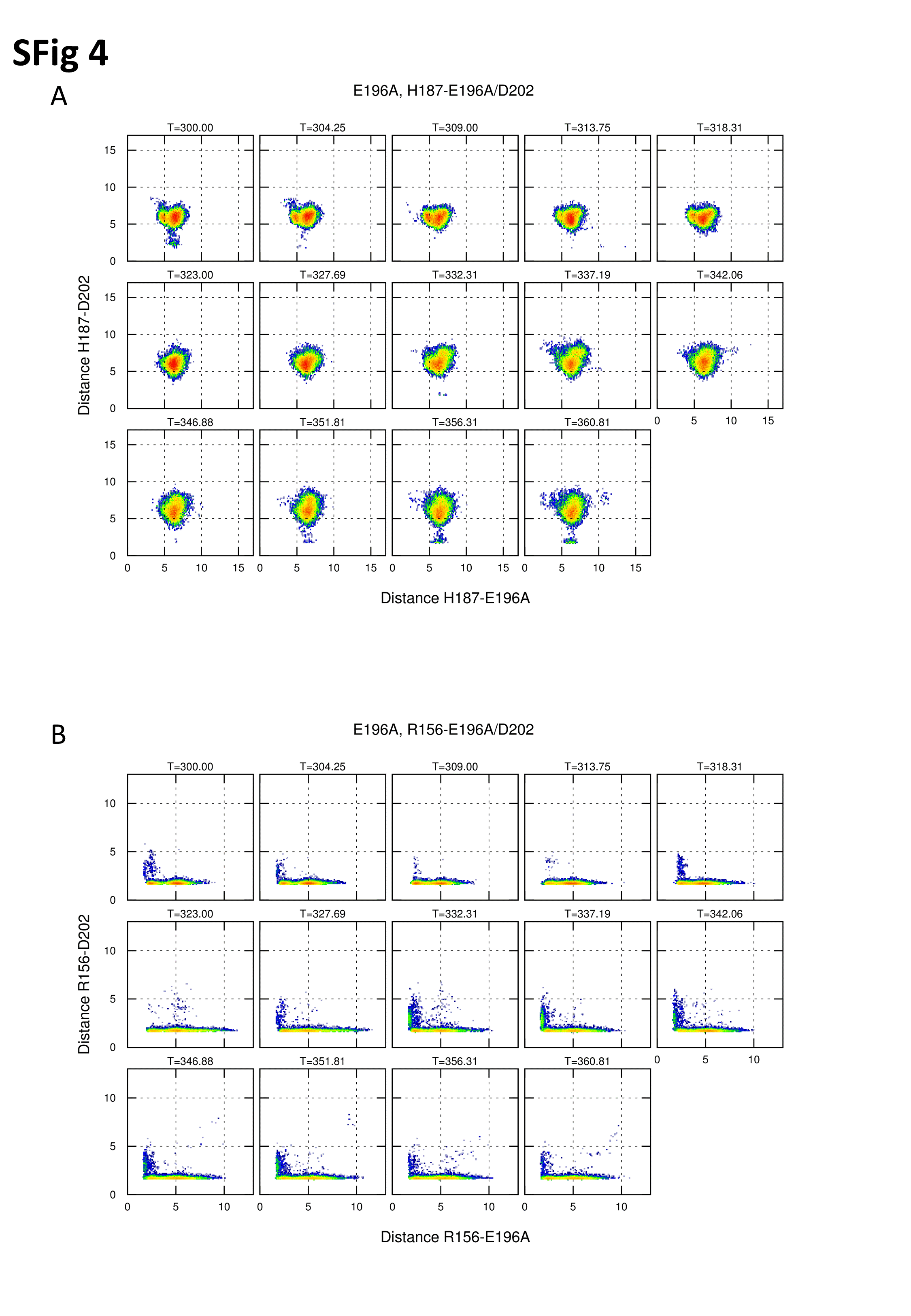


**SFig 4.** E196A mutant conformational free energy heat maps (in arbitrary units) calculated from the populations of the H187–E196/D202 distance (A) and R156A–E196/D202 distance (B) at all temperatures.

**SFigure 5.**


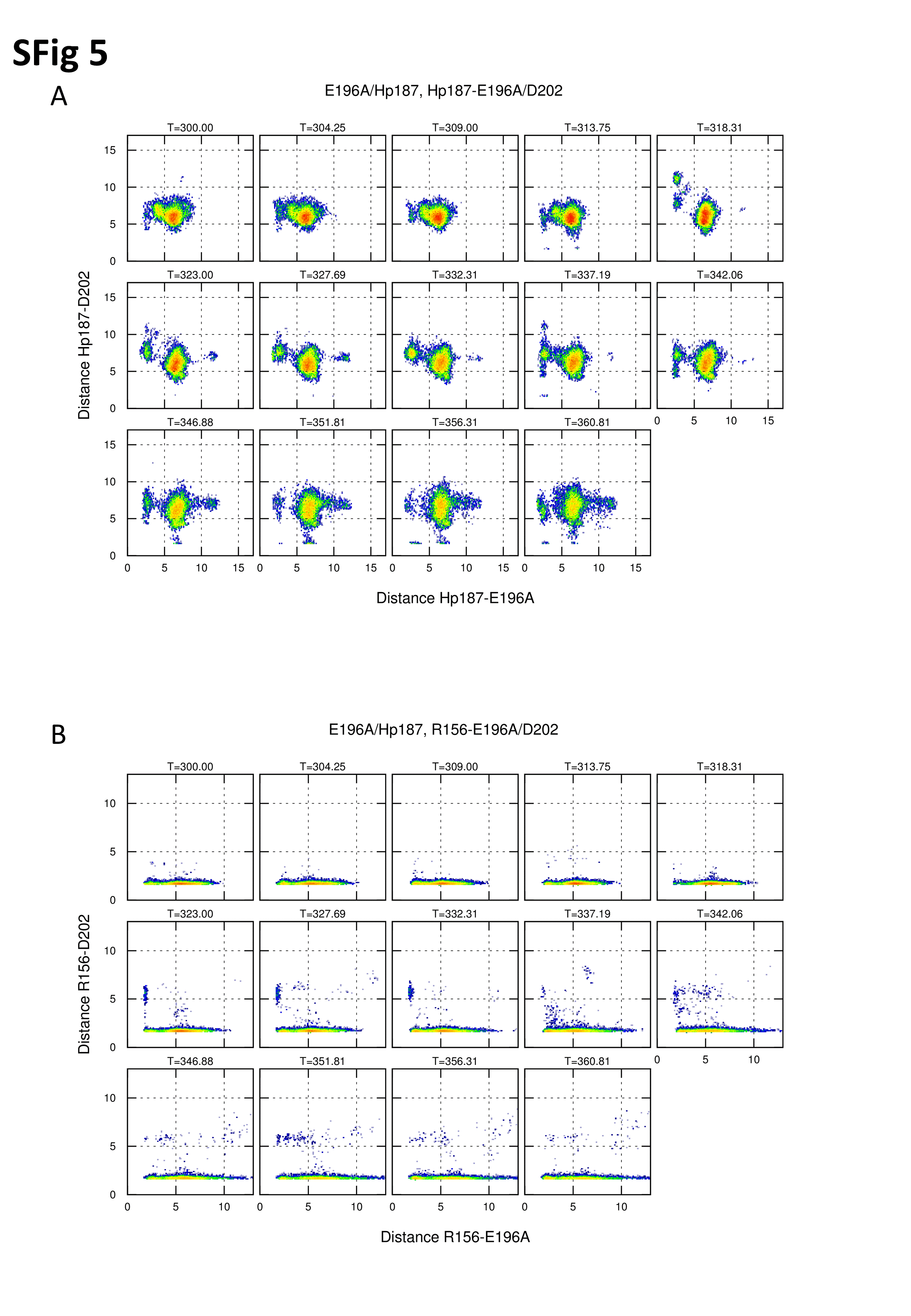


**SFig 5.** E196A/Hp187 mutant conformational free energy heat maps (in arbitrary units) calculated from the populations of the H187–E196/D202 distance (A) and R156A–E196/D202 distance (B) at all temperatures.

**SFigure 6.**


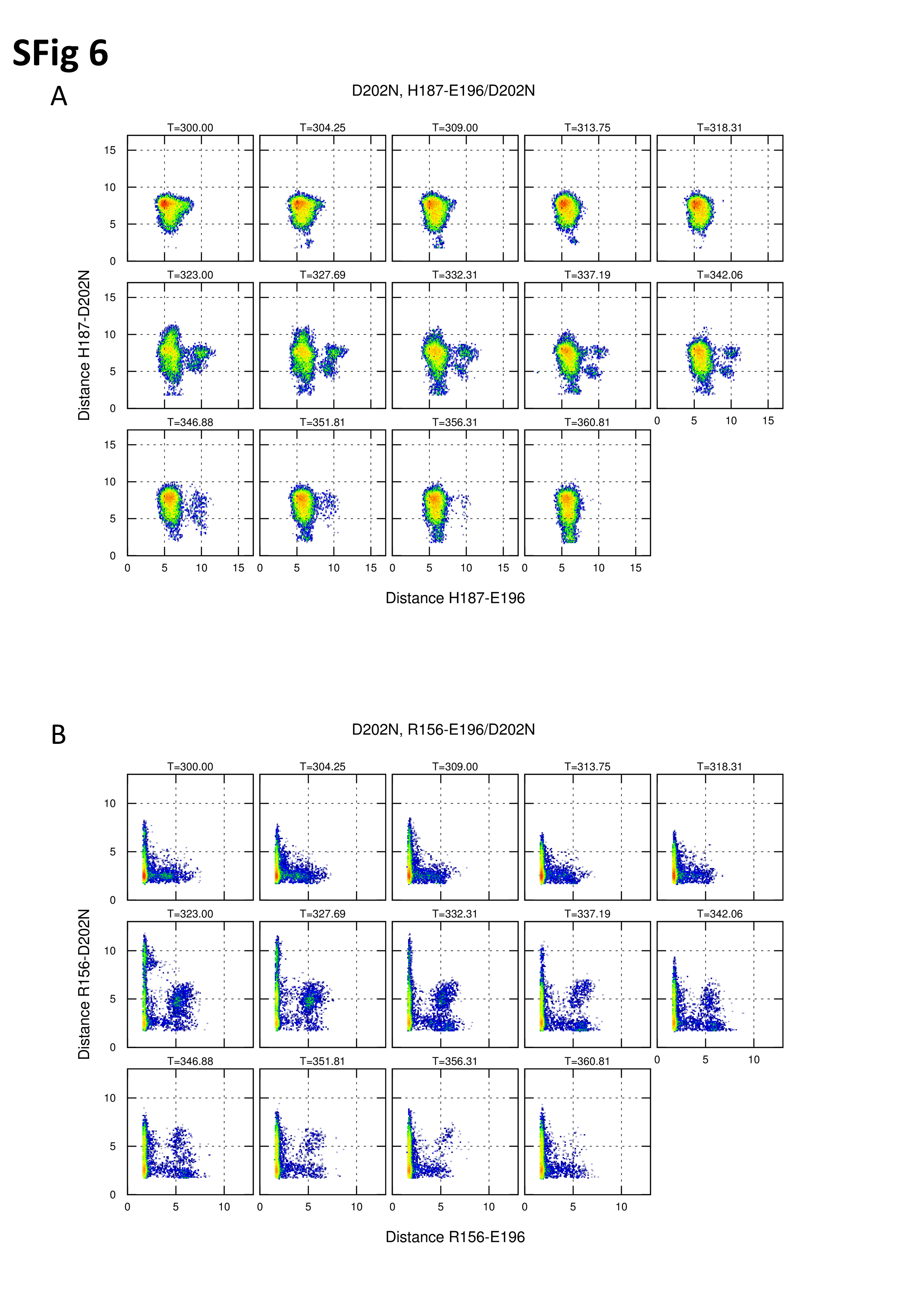


**SFig 6.** D202N mutant conformational free energy heat maps (in arbitrary units) calculated from the populations of the H187–E196/D202 distance (A) and R156A–E196/D202 distance (B) at all temperatures.

**SFigure 7.**


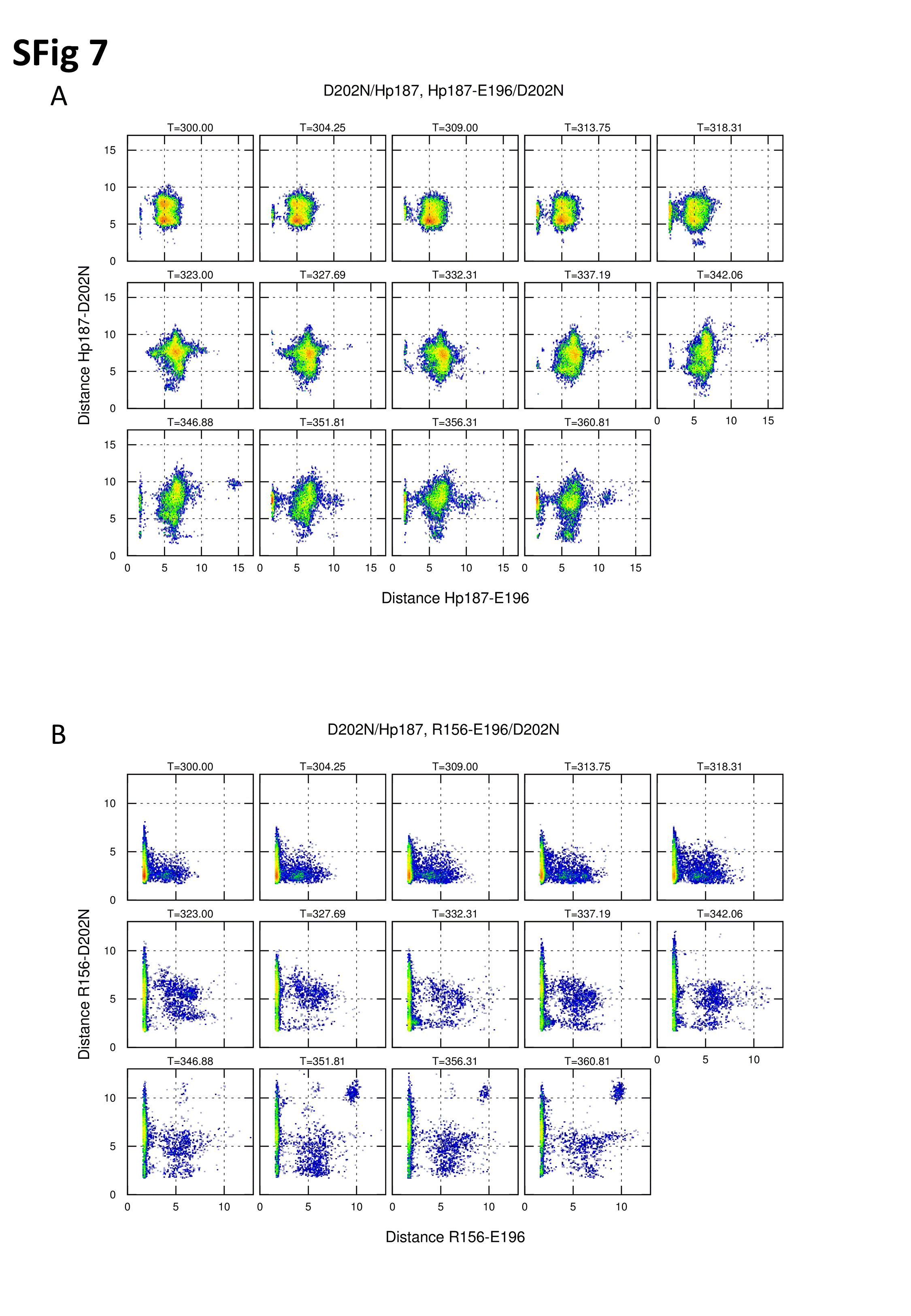


**SFig 7.** D202N/Hp187 mutant conformational free energy heat maps (in arbitrary units) calculated from the populations of the H187–E196/D202 distance (A) and R156A–E196/D202 distance (B) at all temperatures.

**SFigure 8.**


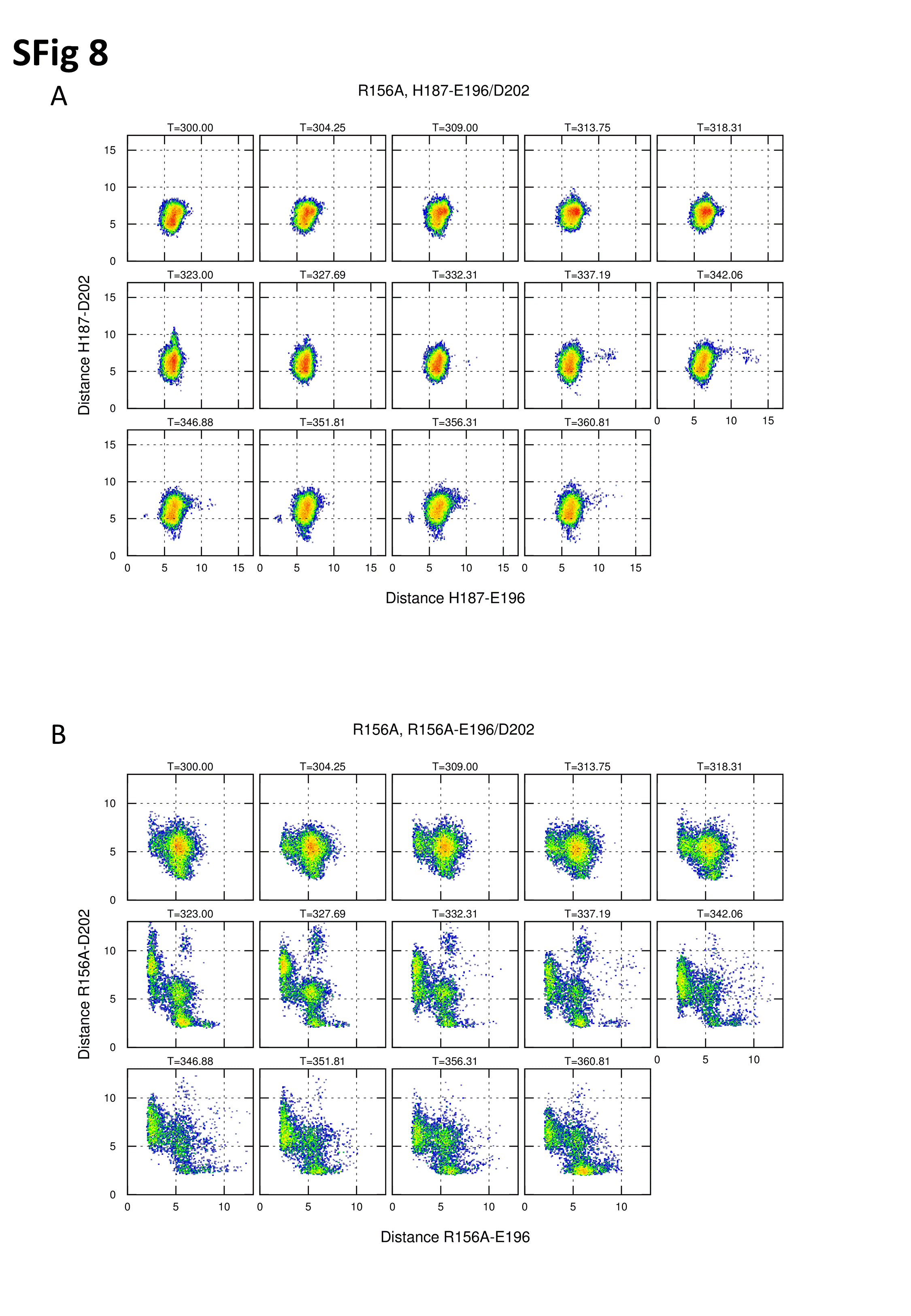


**SFig 8.** R156A mutant conformational free energy heat maps (in arbitrary units) calculated from the populations of the H187–E196/D202 distance (A) and R156A–E196/D202 distance (B) at all temperatures.

**SFigure 9.**


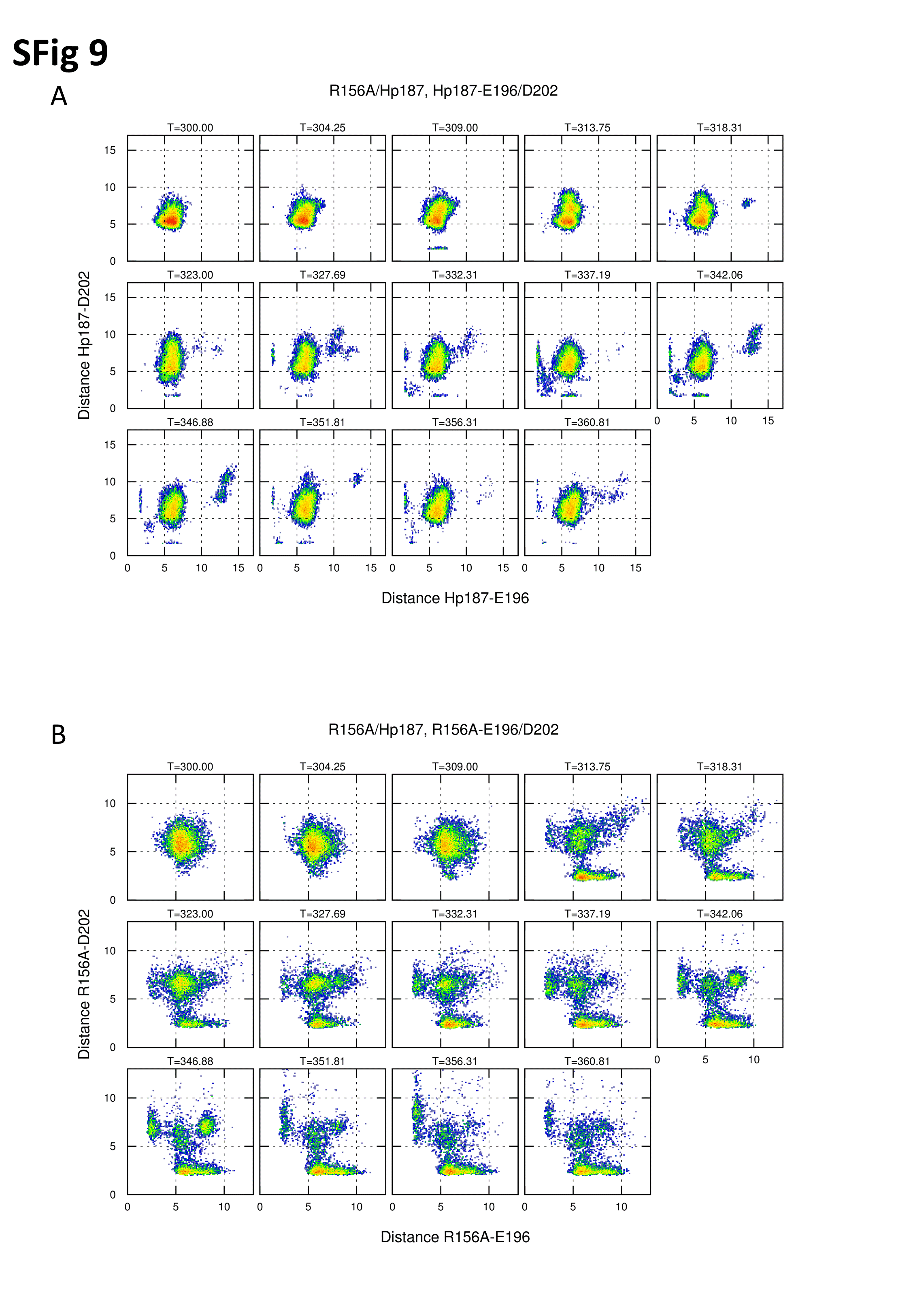


**SFig 9.** R156A/Hp187 mutant conformational free energy heat maps (in arbitrary units) calculated from the populations of the H187–E196/D202 distance (A) and R156A–E196/D202 distance (B) at all temperatures.

**SFigure 10.**


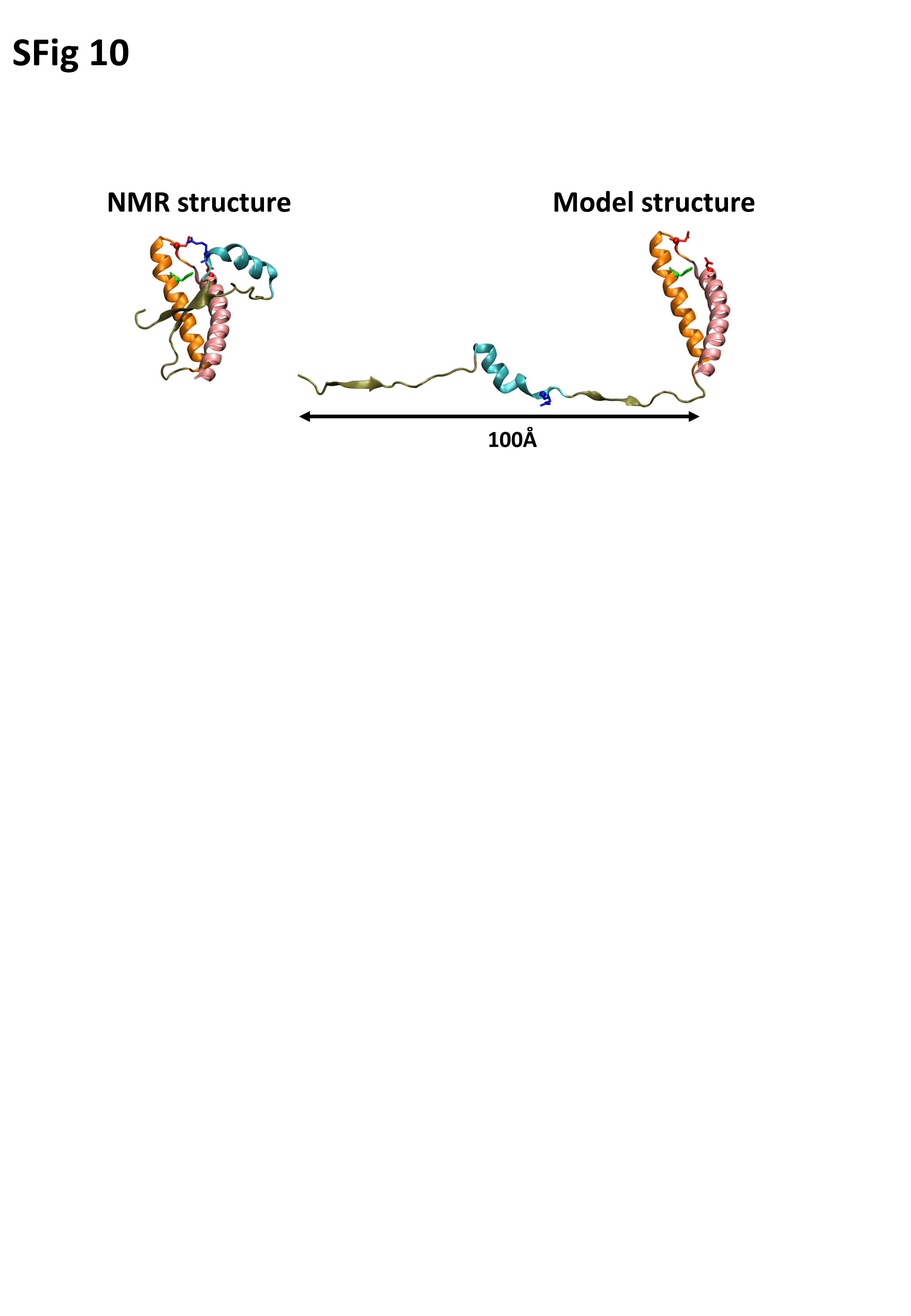


**SFig 10.** The NMR structure of PrP and the model structure with the fully extended fragile part. The model structure was created with the distance restraint between residues 125 and 170.
